# Contraception and post abortion services: qualitative analysis of users’ perspectives and experiences following Zika epidemic in Honduras

**DOI:** 10.1186/s12905-020-01066-7

**Published:** 2020-09-12

**Authors:** Maria Belizan, Edna Maradiaga, Javier Roberti, Maricela Casco-Aguilar, Alison F. Ortez, Juan C. Avila-Flores, Gloria González, Carolina Bustillo, Alejandra Calderón, Harry Bock, María L. Cafferata, Adriano B. Tavares, Jackeline Alger, Moazzam Ali

**Affiliations:** 1 Mother and Child Health Research Department, Qualitative Health Research Unit, Institute for Clinical Effectiveness and Health Policy – IECS, Buenos Aires, Argentina; 2 Instituto de Enfermedades Infecciosas y Parasitología Antonio Vidal, Unidad de Investigación Científica, Facultad de Ciencias Médicas UNAH, Tegucigalpa, Honduras; 3 Department of Obstetrics and Gynecology, Hospital Escuela, Tegucigalpa, Honduras; 4 Centro de Salud Alonso Suazo, Región Metropolitana del Distrito Central, Tegucigalpa, Honduras; 5 Harry Bock. Región Metropolitana del Distrito Central, Tegucigalpa, Honduras; 6 Department of Reproductive Health and Research, World Health Organization, Avenue Appia 20, CH-1211 Geneva 27, Switzerland

**Keywords:** Contraceptive services, Post abortion care, Zika, Reproductive health, Honduras

## Abstract

**Background:**

Zika virus (ZIKV) infection during pregnancy has severe consequences on the new-born. The World Health Organization declared the Zika outbreak to be a Public Health Emergency of International Concern (PHEIC) in 2016. Health facilities in the regions most affected by Zika lacked the capacity to respond to the increased demand for contraception. The objectives were to explore healthcare users’ perceptions regarding contraception, Zika prevention during pregnancy and post-abortion care (PAC) services in the context of a Zika outbreak in Tegucigalpa, Honduras, and to follow these services over time.

**Methods:**

This study was part of a broader implementation research study. We used qualitative research consistent with grounded theory approach. Semi-structured interviews and focus groups were performed with women and their partners who used contraceptive services or received PAC services. Data were collected in two stages from December 2017 to July 2018. Themes explored included contraception, Zika and PAC services.

**Results:**

Participants had positive attitude towards the use of contraceptive methods and demanded more information on safety, efficacy and on side effects. Health care services were inconsistent in the provision of information on Zika and contraception services. ZIKV vector transmission was known but fewer participants were aware of risk of sexual transmission of Zika. Barriers to access healthcare services included contraceptive and PAC services included distance to healthcare facilities, disorganized admission process, long waiting times and out-of-pocket expenditure to purchase medicines. Furthermore, poor quality, mistreatment and abuse of women seeking PAC was prevalent. Some positive changes were noted over time, such as improvements in infrastructure including improved privacy and cleanliness, removal of fees, requisite to bring clean water to hospital.

**Conclusions:**

Our results highlight the challenges and areas for improvement in policy and practice related to contraceptive services and PAC in the context of ZIKV infection. Public policies to prevent epidemics should focus more on providing proper sanitation; removing barriers to access and use of effective contraception as human rights priority. Zika epidemic has highlighted weaknesses in health systems that obstruct access to and use of sexual and reproductive health services.

The study results call for increased efforts to improve access, especially for women of low socio-economic status and intervene at different levels to eradicate discrimination and improve equity in the provision of health care. Qualitative methods can capture the community perspectives and can provide useful information to develop interventions to improve services.

## Background

The Zika virus can be passed from a pregnant woman to her fetus and cause microcephaly as well as other severe brain anomalies, and has also been linked to problems such as defects of the eye and pregnancy loss [1]. The World Health Organization recommended that risk of infection to women of reproductive age should be minimized in affected countries, and that the capacity for surveillance should be strengthened [2]. Moreover, health ministries in Latin American countries had recommended that women postpone or avoid pregnancy, and WHO interim recommendations to prevent ZIKV infection in affected areas included the provision of resources, emergency contraceptive services and counselling [3].

Health facilities in the regions most affected by Zika lack the capacity to respond to the increased demand for contraception [4–6]. Zika has brought the issue into sharp focus and highlights weaknesses in health systems that make it difficult for women to access and use contraception if they want to delay births in the context of widespread Zika infection. In the Latin America and Caribbean region, an estimated 23 million women are not using contraception who wish to avoid pregnancy and around 10% of all maternal deaths are due to unsafe abortion [7–10]. Due to severely restrictive abortion laws, many women may be forced to choose clandestine and unsafe abortion methods [7–9]. Prevention of unintended pregnancy, thus is a primary strategy to reduce adverse pregnancy and birth outcomes related to Zika virus infection. The challenge is that continuation rates for contraception are low in Latin America and Caribbean [3, 7, 11–13]. As contraception and safe abortion are contested issues in most of the countries affected by Zika, it is important that post-abortion care (PAC) is provided. For women who want to avoid pregnancy, access to and use of contraceptive and PAC services is essential [3, 14].

This study is part of broader implementation research study that sought to contribute to the improvement of sexual and reproductive healthcare in Tegucigalpa, Honduras after the zika outbreak in 2016 [2]. The aim of the study was to explore and describe users’ knowledge, perceptions and experiences regarding: Zika prevention during pregnancy, contraception and post-abortion services. As the implementation research made changes during the course of this study, we also aimed to describe any changes in over time.

## Methods

### Study design

The research used a qualitative approach consistent with the grounded theory approach, and following COREQ guidelines to report qualitative research [15, 16].

**Fig. 1:**
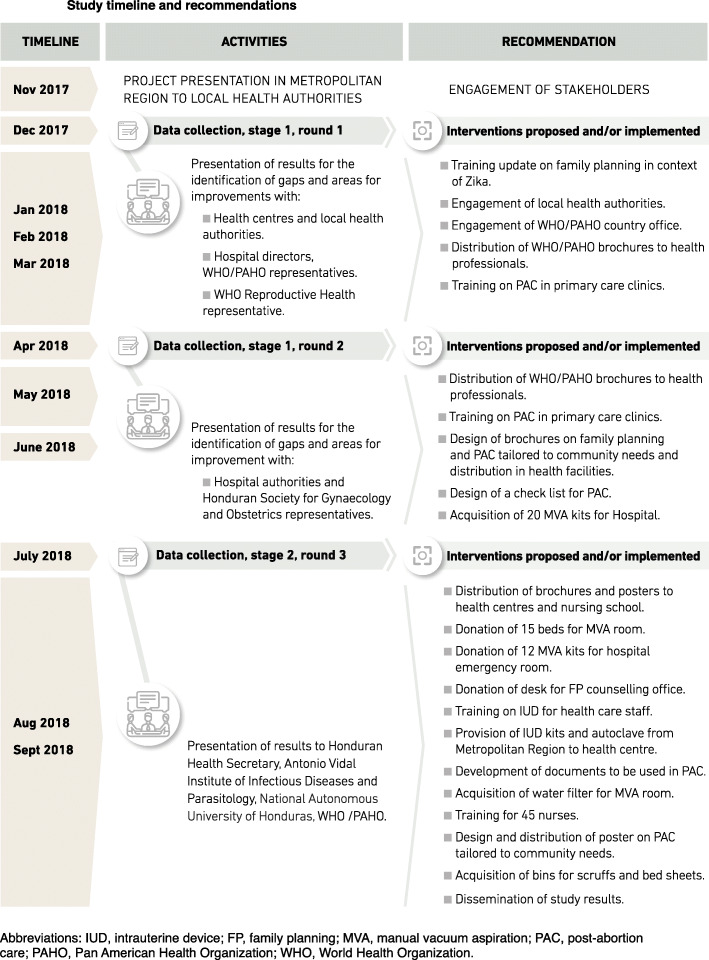
Study timeline and recommendations

This qualitative study is part of a broader implementation research study conducted by World Health Organization (WHO) from September 2017 to August 2018. The protocol of the study was also published [2]. This implementation research study included quantitative and qualitative evaluations and aimed to identify areas for improvement of health care provision for contraception and PAC in Honduras and to implement those improvements, in the context of Zika epidemic in the Americas. As described in Fig. 1, data collection took place in December 2017, April and July 2018. After each evaluation, partial findings were presented to stakeholders including Honduras Secretary of Health, Central District Metropolitan Health Region, the participating health facilities, Honduran Society for Gynaecology and Obstetrics, and World Health Organization/Pan-American Health Organization (WHO/PAHO). Following deliberations, recommendation of interventions for improvement were provided. The results of the quantitative component of this study are reported separately.

### Setting

The study was performed in two public health care facilities in Tegucigalpa, Honduras, corresponding to the Central District Metropolitan Health Region. This 1515 Km2 area has a population of approximately 1.2 million. One of the facilities was a tertiary national general hospital (henceforth Hospital), which provides clinical services, including contraception and PAC; at the time when the study was conducted, it was the only hospital that provided manual vacuum aspiration (MVA) and curettage procedures in Tegucigalpa. -Honduras has only two hospitals with the mentioned characteristics-. The second selected facility was the largest of a total of the 60 health care centres (henceforth Clinic) in Tegucigalpa. The clinic has more than 13,000 consultations per month and provides tertiary health care services, including contraception services.

### Participants and data collection

We performed individual semi-structured interviews and focus groups with quotes of informants including female users of contraception or attending PAC services, male users of health care services, and men who were accompanying partners and relatives with women seeking PAC.

We used a typical case purposive sample to cover three quotes of informants: a) women ≥18 years, users of contraception services; b) men ≥18 years, users of contraception services,) b) women ≥18 years receiving PAC; c) men ≥18 years, accompanying partners and relatives at PAC services. While a minimum number of 5 interviews and 2 focus groups per target group was decided in advance, data collection continued in order to achieve the saturation criteria.

Questionnaires were developed based on the study objectives and included the following themes: (1) Contraception: knowledge and attitude regarding contraception use, opinions about quality of contraception services; (2) Zika: knowledge about transmission of disease, risks and consequences during pregnancy, and practices for prevention strategies during pregnancy; and (3) PAC: access and use, users’ experiences, opinions about quality of care.

The questionnaire and interview guide were piloted and adapted to local context (See additional files). The interviews and focus groups guides developed for this study are provided as Additional File 1. Data were collected in three rounds. During the first round in December 2017, the three themes were explored (Contraception, Zika and PAC). The second round in April 2018 was used to obtain a deeper understanding of preliminary findings of the first round regarding PAC. In round three in July 2018, all themes explored in previous rounds were included in order to identify any change. Interviews and focus groups guides were adapted to each round.

Female trained data collectors interviewed women and male trained data collectors interviewed men. Individual interviews lasted between 30 and 50 min whereas focus groups took about 60 to 110 min. All participants gave their written informed consent respectively. All interviews and focus groups were audiotaped and transcribed verbatim.

### Ethical aspects

Following approval by WHO scientific and ethics review, the protocol was reviewed and approved by the IRB at the Faculty of Medical Sciences of the Universidad Nacional Autónoma de Honduras (UNAH), Tegucigalpa, Honduras (Reference Number: 201750). All participants were informed about the objectives of the study and their rights, and were provided a written informed consent. Study forms were numbered, and no individual personal information or identifiers was entered in the audiotape and written transcripts.

### Data analysis

The written transcripts were entered into ATLAS.ti version 8.1 (Scientific Software Development GmbH) qualitative data management software. Using adapted grounded theory and thematic analysis, transcripts were coded line by line, and three investigators inductively identified concepts. Then, matrices were developed to facilitate comparisons across the transcribed materials and to retain the context of the data (i.e. clinic, and type of informant). To ensure that coding captured all relevant issues and reflected the primary data, thematic analysis was shared with all the data collectors to discuss the findings. Finally, data was abstracted and interpreted. Illustrative quotes were included. The description of the findings is organized in sections to address contraception, Zika and PAC services. We included examples indicating between parentheses the technique used (Focus Group or Interviews), sex of informant (woman or man) and the facility (Hospital or Clinic) where interviews took place.

## Results

### Population

During the first stage (first two rounds of interviews) five focus groups and 36 individual interviews were performed. In the second stage (round three), five focus groups and 21 individual interviews were performed.

A total of 136 individuals participated in the study: 85 (62.5%) women and 51 (37.5%) men; median age was 26 (range 19–44) years for women and 29 (range, 19–59) years for men; 77 (56.6%) participants had completed secondary school (Table 1). Approximately 5% refused to participate in the study mostly citing personal reasons and lack of time.

**Table 1:** Characteristics of the participants interviewed and focus groups, Tegucigalpa, Honduras

Characteristic	Composition of study population (*n* = 136)
**Gender, n (%)**	
Female	85 (62.5)
Male	51 (37.5)
**Age, median (range**)	
Women	26 (19–44)
Men	29 (19–59)
**Education level, n (%)**	
Primary	40 (29.4)
Secondary	77 (56.6)
University	16 (11.8)
None	3 (2.2)
**Current work status, n (%)**	
Working out of household	70 (51.5)
Unemployed	66 (48.5)

The result sections are organized to highlight issues in contraception, ZIKV infection and PAC services, respectively.

### Contraception

#### Knowledge on contraceptive methods

Most participants knew at least one contraceptive method. The most frequently mentioned were pills, injections and implants, while less frequently mentioned methods were intrauterine devices (IUD), male condoms and surgical sterilisation. Both female and male participants knew about female condoms but lacked detailed information about them. Both men and women said that although they knew they could choose different contraceptive methods, they would like to receive more detailed information on each individual method, specifically regarding their adverse effects and efficacy.

“*They don’t provide advice regarding family planning and they only ask “What kind of family planning do you want?” without informing the patient about the side effects of the different methods, for example the risks that an IUD can bring in the future, or the risks of the injection, so I end up choosing the method that a friend recommended*”. (Focus Group, women, Hospital).

#### Attitude towards contraception

Participants generally had a positive attitude towards the use of contraceptive methods. Women and their partners said they did not feel stigmatized for seeking information on methods at the health facilities. Few men acknowledged they did not allow their partners to use contraceptive methods, while others considered contraceptive use should be a shared decision. Despite the knowledge about contraceptive methods and the positive attitude, many participants specified that their children had not been planned. In fact, many women said that they had sought contraceptive methods only after giving birth.

#### Concerns about contraceptive methods

Many participants shared doubts about the effectiveness and the adverse effects associated with contraceptive methods and had concerns on the lack of comfort when using them. Most participants believed that the effectiveness of each contraception method varied with each person. In fact, women thought that although physicians had information on the methods, they may not know how individual women’s bodies would react to each method. Many participants provided anecdotes involving the experience of friends or family that supported their perception that any method, even surgical sterilisation, could fail.

“*This guy had sex with a lady, and she got pregnant, and he said it was not his baby …. And then there was a fight at home. When he came to the hospital he got examined and he really was fertile even though he had been operated! Could you just imagine if he had killed that woman!*” (*Focus Group, Women, Clinic*).

Women most commonly accepted and used hormonal contraception; indeed, many preferred the three-month injection because it was freely distributed at the public hospital and was easily accessed. Participants’ main concerns regarding hormonal methods were their efficacy and their association with cancer, infertility, weight gain and skin blotches.

“*I am scared of implanon [implant], I’m honest. No doctor will tell you the truth, they tell you the implanon will cover this or that, but they won’t tell you that in the future it will give you cancer*”. (*Focus Group, Women, Clinic*).

Pills though had a burdensome daily regimen, were considered a very effective method. Participants had information on morning-after pills but explained that the use of this method was frequently associated with rape. For men, the most popular method were male condoms; however, they would use it for the prevention of sexual transmitted diseases (STI) rather than for contraception. Of note, only a few participants included Zika in the group of diseases that could be prevented through condom. Some male participants were aware of surgical sterilisation (vasectomy), although only very few men were considering having it.

#### Barriers in access, distance and hospital hours

Participants observed that opening hours at health centres were limited and that the distance between their homes and the health facilities was a significant access barrier. Therefore, participants often arrived at the hospital at 4 am to secure an appointment. Most of the participants said that the admission process was disorganized and difficult and that waiting times were very long and thought the probable cause of delays was insufficient numbers of staff and lack of administrative organization.

*“Look, I came here on Sunday, at 9 am and they told me I had to come back at 1pm. I came back at 1pm and they told me they were not open because they were going to disinfect the place. So, I came back on Monday and told me to stay on a line of people, I tried to explain to the girl, so they told me to return in the afternoon”. (Interview, Woman, Clinic).*

Regarding infrastructure, some participants complained that the toilets were not clean; however, at the last round of survey, a few participants acknowledged there had been some improvements in cleanliness and infrastructure.

#### Quality of services

Participants considered that counselling services on reproductive health, especially on contraception were inconsistent and insufficient. Those who wanted to use contraception, received information provided at compulsory meetings only. Several participants suggested that health professionals should proactively approach people and give them more information on reproductive health and that counselling service should be extended to all even during the afternoons and evenings.

*“The service here (public health centre) is not the same as in a private clinic where you have to pay because here there are so many people. The doctors have to see so many people that they cannot have a special relationship with you” (Focus Group, Women, Clinic).*

#### Privacy and confidentiality

Some women felt uncomfortable during the consultations due to lack of privacy. They would prefer female providers.

*“I don’t know why, but with women we have more confidence, though some men are more sensitive sometimes, but for intimacy issues it is better to have a woman [as health personnel]”. (Focus Group, Women, Hospital).*

Often, single women sought counselling service and information in clinics outside their communities to avoid the embarrassment of being recognised by neighbours or healthcare staff. Moreover, it was reported that, sometimes, confidentiality was ignored by health staff, and this contributed to users’ mistrust in the services.

*“You know what I’d change? I’d make this office private for women who come here for the (DMPA) shot, because you have to pull down your trousers, and you really feel embarrassed, sometimes in presence of three, four, five other people there” (Focus Group, Women, Clinic).*

#### Access to methods

Participants stated that they could receive some contraception methods free of charge, including three-month injection, condoms and IUD. However, participants perceived that the condoms freely distributed at health facilities were small and of bad quality; therefore, when needed, they preferred buying at the pharmacies. Those condoms were considered to be inexpensive, of better quality and accessible in nearby vicinity. No improvement in services were perceived by the participants throughout the interview rounds.

### ZIKA virus infection

#### Transmission of zika

Participants were aware that Zika was mainly transmitted by mosquitoes. Participants mentioned that pregnant women could be infected if they had sexual intercourse with an infected person and that this could be avoided using condoms.

*“I know that (Zika) is transmitted by the mosquito, that is the main thing, and that we have to pay attention if we have the basins, if we have plants and barrels, we have to keep them clean to avoid larvae and mosquitos at our houses” (Focus Group, Women, Hospital).*

However, from rounds one to three of survey, the number of participants who also knew of the possible sexual transmission of Zika, decreased (in correlation with distance in time with the outbreak).

#### Risks involved in zika

Participants mentioned that Zika infection was associated with serious risks for pregnant women. Most mentioned that the baby could get sick, die in the womb, have problems in the head and brain; suffer from microcephaly. Fewer mentioned the Guillain-Barré syndrome and malformations. There was confusion among participants about the difference among Dengue, Chikungunya and Zika infections. For them, the three diseases had the same symptoms and consequences in men or in non-pregnant women. This perception didn’t change in the different rounds of interviews.

*“Well, I’ve heard that it affects people, pregnant women, and the baby. When someone is infected, they can infect the baby and cause complications” (Focus Group, Women, Hospital).*

“*We must be careful, if we are pregnant, we should be especially careful of not getting a bite, because the baby could be affected”. (Focus Group, Women, Hospital).*

#### Sources of information on zika

Most frequently mentioned sources of information were television and radio, followed by Facebook and informative posters at health facilities. During round one of data collection, participants recalled having received information through mass or social media on how to avoid or control mosquitos at home and recalled having seen images of babies with microcephaly.

*“What I know is how to prevent the disease, but not how to take care of oneself once you get the disease; most of all I know about prevention, that includes a clean household and getting rid of all possible mosquito breeding places and that is what they mainly talk about in the news” (Focus Group, Women, Hospital).*

Health professionals were the most reliable source of information. However, at the last round of survey, at the end of epidemic, fewer participants received information or noticed any brochures or posters at the health facilities.

*“Information on Zika is only available when there is an outbreak, they give information to prevent the disease. Why is information available only during outbreaks?” (Focus Group, Men, Hospital).*

#### Prevention of Zika

Participants mentioned that they knew of some preventive measures mostly related to the vector transmission; such as covering the water tank (called pila) and cleaning it; however, the information on the frequency of cleaning was not clearly communicated.

*“We should wash well the sink with chlorine, if you see a tire there with water you must empty it, if you have a leak, or containers full of water they have to be emptied, because that is where the mosquito comes from, wash all the ditches, everything has to be cleaned as much as possible*” *(Focus Group, Women, Hospital).*

They knew to use repellents, mosquito nets and coils, and cut tall grass and bushes. Less frequent practiced methods were covering buckets, cleaning gutters, or using a fan to repel mosquitos. Aside from the measures they knew of, participants explained they cleaned their house, and used larvicide and bleach in water tanks. However, many participants were against burning mosquito-repelling incense indoors because the smoke caused cough. Of note, by the third round of interviews, participants stressed that actions performed by public and technical teams in the prevention of Zika in neighbourhoods had ceased.

#### Healthcare seeking behaviour for Zika infection

Upon suspicion of Zika, few participants sought care at a health centre and were referred to a hospital to confirm the diagnosis. However, not everyone attended a health centre; some were diagnosed by a relative who knew of the disease and its symptoms. Moreover, these patients acknowledge they were self-medicated with acetaminophen and liquids, because they knew how physicians treated other cases of Zika infection.

### Post-abortion care (PAC)

**Fig. 2:**
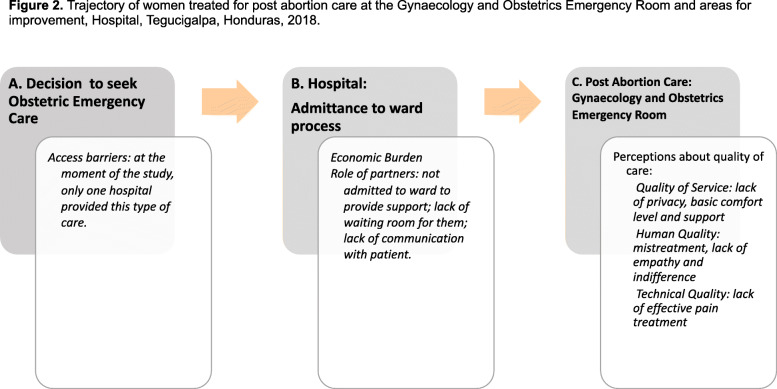
Trajectory of women treated for post abortion care at the Gynaecology and Obstetrics Emergency Room and areas for improvement, Hospital, Tegucigalpa, Honduras, 2018

The process for women receiving PAC services at the Gynaecology and Obstetrics Emergency Room at the hospital is described in Fig. 2.

Women seek emergency obstetric care because either they are referred by a health centre or hospital to manage symptoms such as bleeding or pain; or because they are following up treatment with the drug misoprostol (A). Someone (partner, relative, etc.) may or may not accompany them to hospital (B). Those accompanying the women are not allowed inside the premises of the hospital. At the entrance of emergency gate, women are searched by security personnel and then escorted to a waiting area; those who are not ambulatory are assisted by hospital assistants in wheelchairs. In the waiting area, medical students interview the patients and refer them to specific services. Women needing PAC services are admitted to the Gynaecology and Obstetrics Emergency Room (C). Women in emergency are attended round the clock and those with suspected pregnancy are offered pregnancy test (rapid test).

Physicians register patients’ data and this registry is completed and collected daily by the statistics department. Misoprostol is prescribed and ultrasound is performed on all women. Patients under observation receive family planning counselling in the same room.

#### Geographic and economic challenges in accessing PAC services

Women when referred, are warned that reaching hospital could take them up to two hours, or even more if transportation was unavailable. Participants complained that no other public hospitals offered PAC and highlighted the need for these services to be closer to their homes.

*“I decided to come today because I was bleeding so much, I fainted, had cold sweats, nausea and my body was shaking” (Interview, Woman with MVA procedure, Hospital).*

Women had to cover the transportation cost to the only hospital that offered PAC services in the city, and had to pay 200 Lempiras (approx. US$8) per treatment. However, it could reduce to 50 Lempiras (approx. US$2) if social services office certified that the patient could not afford the full fee. Moreover, the hospital also required the patients to bring a gallon of water to clean the medical equipment.

Some patients had to pay for their medication and most women paid for a rapid pregnancy test. It was mentioned that misoprostol, anti-inflammatory agents and analgesics were not freely available; instead, patients had to buy them out-of-pocket. Many times, these payments for specific treatments were out of reach of patients.

*“They asked me to buy a pregnancy test. Afterwards when they were about to perform the MVA they told me that I had to pay 200 Lempiras and purchase a container of water”. (Interview, Woman, Hospital).*

By the final round of this survey, following discussion of research team with health authorities, this requirement and the fee was eliminated. The hospital administration purchased a water filter system for emergency room and medical staff with research study’s funds.

#### Absence of waiting space

Participants complained that male partners and relatives were not allowed into the hospital and had to wait long hours without any protection from the weather; slept on the floor at night and received no updates on the health of their partners from physicians or nurses.

*“The cleaners just started to throw water on the floor, they didn’t mind if I had my clothes there or if I was sitting there. I was sitting there, after two nights without any sleep, I fell asleep on my bags on the floor and when I woke up because someone told me to, they were throwing water at me” (Interview, Man, Hospital).*

Women seeking for emergency care remained unaccompanied in the waiting area and during the procedure.

*“Through emergency, I got her [wife] in and the guard treated me badly because I came with her and he told me that I couldn’t go in and I told him that it was an emergency because she was my wife and she was really sick and had problems”. (Focus Group, Men, Hospital).*

#### Insufficient infrastructure for services

A very common complaint during the first round was the lack of privacy for the women who sought assistance or during a procedure, including the lack of sufficient beds or rooms. Most participants complained that the admission process was disorganised, difficult and time-consuming, the public toilets were unclean, and medications were often lacking.

The reproductive health counselling was only given after MVA procedures were performed, with no privacy and in the presence of other patients. Over time, participants acknowledged some improvements specially ward cleanliness.

#### Mistreatment and abuse

Many participants agreed they had been mistreated by physicians and nurses when seeking PAC or emergency care. Some of the women felt that they were laughed at, reprimanded and ignored by both physicians and nurses.

*“Today, I was undergoing the MVA, the doctor was doing the procedure with people standing by the door, there was a student asking one thing and another and doctor’s phone kept ringing”. (Interview, Woman, Hospital).*

The most common complaint was the staff’s lack of empathy and indifference to their health situation.

*“My mom asked for information because when I was in the ward and there were many girls who had had an abortion and they told me ´you have to rest, you can’t crouch, you can’t travel´. So, I told mom to get information. The first time I had an abortion, they didn’t explain anything to me, they just told me ´go away and take care!´, just that. So, my mom asked a nurse and she just said I had to rest, just that” (Focus Group, Women, Hospital).*

Accompanying male partners and female patients described instances of mistreatment by the security staff when being admitted to the hospital. The male partners mentioned that they did not receive information about the patients’ health and procedures during in-hospital stay, the remarked they feel mistreated and that produced distress and fear.

*“My partner was being accompanied by her sister. I could not get in touch with my partner, I could not even call her on the cell phone. Then, I could talk to her sister, she called me, she was crying and told me that my baby’s heart was not beating and the doctors had performed an ultrasound and had seen my baby was dead and that they going to do another ultrasound” (Interview, Man, Hospital).*

#### Quality of services

Some women complained of having to wait for long hours in pain, only to receive what they perceived as unsatisfactory care. Some women who were prescribed misoprostol felt that they had been discharged without adequate information as they felt staff did not sufficiently explain the procedures to them. Some patients reported that physicians did not treat and even ignored the symptoms they complained about, in particular, pain. After unsuccessful treatment in their own home, some participants had to return to hospital for treatment.

*“It is a horrible sensation, because one feels as if they are pulling the uterus and the doctors only tell us to cooperate but that is, it is impossible to stay relaxed, when one is going through something so painful.” (Interview, Woman, Hospital).*

## Discussion

The qualitative approach used in this study captured community perspective regarding contraception services, and quality of PAC in the context of the Zika virus outbreak in Tegucigalpa, Honduras. Our findings contributed to identify improvement areas and to implement solutions. The study shows particularly unmet needs for contraceptive counselling and access to methods. Women and men of reproductive age demanded further information on contraceptive methods; in addition, we found structural and cultural barriers to access to sexual health care still exist.

Participants had information on the risk of Zika during pregnancy and were aware of the vector transmission. Interestingly, participants perceived that Zika, Dengue and Chikungunya were different stages of the same disease. Sources of information mentioned by participants about Zika included mass media, social media, other community members and healthcare providers. Paradoxically, physician and nurses were perceived as the most trustworthy sources of information. By the final round of the study, fewer people could identify the risk of sexual transmission of Zika, and participants noticed that less information on the disease was available and acknowledged that preventive actions by governmental technical teams had ceased. Similarly, it has been reported that knowledge about sexual transmission was low among Brazilian women [17].

Findings also indicate the existence of geographical and economic barriers to accessing healthcare services, poor quality of care and mistreatment of women seeking PAC and for their partners. The improvement strategies implemented by health authorities based on the partial findings of the study were acknowledged by healthcare users; participants mentioned that the consultation fee and the requirement to bring clean water to the health facility had been eliminated. Comparable results were found in pregnant women seeking healthcare during Zika epidemic in Colombia, where out of pocket payments and insufficient information acted as barriers [18].

Current modern contraceptive methods available in Honduras were mentioned by study participants: contraceptive pills, condoms, intrauterine device, sterilization, injectables, hormone implants, patches, diaphragms, spermicidal agents, and emergency contraception. Coinciding with previous reports [11, 19], women were knowledgeable and supportive of a range of contraceptive methods. However, many informants revealed that their pregnancies had not been planned. It may be the case that larger structural factors prevented them from obtaining their method of choice [11]. It was also found that Honduran women frequently changed contraceptive methods, suggesting women encountered problems with the methods and that they changed their pregnancy intentions [20].

As seen in this study, anecdotal information plays an important role in choosing a method and in deciding to seek medical help [21]. Self-medication and shared information instead of medical care are common in other Latin American countries [22]. In low income countries, women, in particular adolescents, still resort to dangerous solutions when legal abortion is not available, indicating current system and structural failures inherent in service systems [12]. As in the case of Brazil [17], there seems to be a missed opportunity to counsel women about reproductive health when they use emergency care or when they visit a health facility. Information sharing norms exist around sexual and reproductive health [12]. In the past years, due to the support by international organizations, Honduras has been implementing family planning programs to improve contraceptive supply chains and information flows [19].

The ZIKV infection still poses a public health threat and the huge population living in *Aedes*-infested regions makes re-emergence of ZIKV infection likely; therefore, it is critical to improve health system capacity and increase efforts in LMIC to respond to future Zika epidemics [23]. In our study, problems on the quality and accessibility of PAC services seemed to be independent of the ZIKV epidemic; however, the increased need of maternal healthcare in the context of an epidemic may make those problems more evident. Some structural problems in facilities were addressed during the research study, and, indeed participants acknowledged and appreciated the positive changes. For example, consultation fees for women seeking care were reduced or eliminated; the requirement to bring clean water to the hospital was eliminated. Other improvements at the end of the study included the printing and distribution of brochures and posters about contraceptive methods and their failure rates; redistribution of furniture and functionality at the MVA ward to transform a staff resting room into a clinical examination room to provide privacy; addition of FP counselling services; and training of staff on humanized PAC.

Many countries with autochthonous Zika transmission have restrictive abortion laws, particularly affecting women of lower socioeconomic status. Indeed, in Honduras, abortion is illegal in all circumstances; therefore, legal and functional restrictions block this option for women without economic means and for those whose choices are restricted by geographical access [24]. At the time of the data collection, women who experienced an abortion could receive evidence-based care in only one public hospital of Tegucigalpa. For this reason, the first main barrier was geographical access; women who did access healthcare also faced problems in the admission process.

Participants described their experience when seeking health care as physically and emotionally disturbing. Institutional violence during PAC was mentioned by many participants; comparable experiences were described by women in Brazil, another country that experienced Zika outbreak [25]. Participants reported discriminatory practices, moral judgement, negligence in the control of pain, lack of privacy, and long wait for uterine curettage. Privacy is a key element in quality of care and a basic human right. Women’s accounts suggest this is neglected in healthcare centres, where privacy is violated in situation of vulnerability.

In the study, violations of autonomy and informed choice were also described by women in which seems to be a culture of asymmetrical power relationships; a usual reality for women who are affected by Zika and other public health crisis, in which women find it difficult to freely decide about their lives, their sexuality, their reproduction and their bodies [14]. In a recent nationally representative study among Honduran women, physical, sexual, and emotional interpersonal violence were associated with sexual and reproductive health outcomes; indeed, contextual factors such as gender inequality may contribute to this in this context [26]. In our study, women reported instances of harmful practices not only within the health system but also outside. Authorities still need to address the imperative problem of ill-treatment. Interventions to improve access and adequate healthcare may include policy, health service structure, and continuous training, among others. It has been shown that interventions in human resources for health impact positively on improving maternal health [27]. It is also necessary to provide education, alleviate poverty, and improve infrastructure, establish gender equality among other steps for improved maternal health [27].

During the Zika epidemic, women in our study did not mention having received specific information on Zika when they used emergency care and that information on counselling was inconsistent; in fact, information seems to be distributed only during specific campaigns against diseases such as Zika or dengue. Counselling at health facilities is a crucial intervention for the promotion of contraceptive use. However, as seen in our study, visiting a health facility per se does not necessarily mean that a woman would receive counselling [19]. For community members with lower education levels (as the participants included in our study), awareness campaigns may be useful. This is a strong message in itself. Coinciding with another report from Brazil [17], women in our study were not fully aware that Zika could be sexually transmitted and counselling about this issue was limited. Preventing the spread of Zika virus is very important but it is also necessary to combat congenital Zika syndrome epidemic through access to contraception, education, and access to safe abortion services [28]. Clinicians have a role in ensuring that patients receive information to obtain reproductive health care, which includes management of post-abortion complications [29].

### Strength and limitations of the study

This qualitative study succeeded in the inclusion of typical cases of health care users and their companions. Given the nature of the study design, the sample of participants is not representative of the larger population of the healthcare users in the country. Instead, we explored thoughts, opinions and experiences of purposively selected population. We included users of the two main health facilities providing reproductive health services and the only health facility providing PAC in Tegucigalpa at the time of the study. Users from rural areas were not represented. The results of the study do not mean to be generalizable but contributes to know and understand the healthcare circumstances from the users point of view. There is the possibility that some participants reserved responses for fear of scrutiny from investigators or peers. However, all participants contributed to the discussions. Although results of the research project from which this study stems had implications on how Zika epidemic in Honduras was addressed, the timeframe did not allow for measuring changes adequately. This study is an example of implementation research in low and middle-income countries and its benefits by facilitating the delivery of evidence-informed interventions [30].

## Conclusions

Our findings highlight the challenges and areas for improvement in policy and practice related to contraception services and PAC in the context of ZIKV infection. The methodology allowed us to identify and record the community perspective. Zika epidemic has highlighted weaknesses in health systems that obstruct access to and use of sexual and reproductive health services. Public policies to prevent Zika epidemics should focus more on providing proper sanitation and removing barriers to access and use of effective contraception.. The study results call for increased efforts to improve access, especially for women of low socio-economic status and intervene at different levels to eradicate discrimination and improve equity in the provision of health care.

## Supplementary information

**Additional file 1.**

**Additional file 2.**

**Additional file 3.**

## Data Availability

The qualitative data will be available upon request. Requests can be sent to alimoa@who.int

## Abbreviations

FP: Family planning
LAC: Latin America and Caribbean
LMIC: Low and Middle income Countries
MVA: Manual Vacuum Aspiration
PAC: Post abortion care
PAHO: Pan-American Health Organization
PHEIC: Public Health Emergency of International Concern
RH: Reproductive Health
SDG: Sustainable Development Goal
STI: Sexually Transmitted Infections
WHO: World Health Organization
ZIKV: Zika Virus

## References

[1] Petersen LR, Jamieson DJ, Powers AM, Honein MA (2016). Zika Virus. N Engl J Med.

[2] Ali M, Folz R, Miller K, Johnson BR, Kiarie J (2017). A study protocol for facility assessment and follow-up evaluations of the barriers to access, availability, utilization and readiness of contraception, abortion and postabortion services in Zika affected areas. Reprod Health.

[3] Darney BG, Aiken AR, Kung S (2017). Access to contraception in the context of Zika: health system challenges and responses. Obstet Gynecol.

[4] Harris LH, Silverman NS, Marshall MF (2016). The paradigm of the paradox: women, pregnant women, and the unequal burdens of the Zika virus pandemic. Am J Bioeth.

[5] Schuck-Paim C, Lopez D, Simonsen L, Alonso W. Unintended pregnancies in Brazil - a challenge for the recommendation to delay pregnancy due to Zika. PLoS Curr. 2016;8. 10.1371/currents.outbreaks.7038a6813f734c1db547240c2a0ba291 PMC4866532 28515967

[6] Diniz D (2016). Zika virus and women. Cad Saude Publica.

[7] Ali MM, Cleland J (2005). Sexual and reproductive behaviour among single women aged 15-24 in eight Latin American countries: a comparative analysis. Soc Sci Med.

[8] Diniz SG, d'Oliveira AFPL, Lansky S (2012). Equity and women's health services for contraception, abortion and childbirth in Brazil. Reprod Health Matters..

[9] Darney B, Aiken A, Küng S (2017). Access to Contraception in the Context of Zika. Obstetrics Gynecol.

[10] Roth GA, Abate D, Abate KH, Abay SM, Abbafati C, Abbasi N (2018). Global, regional, and national age-sex-specific mortality for 282 causes of death in 195 countries and territories, 1980–2017: a systematic analysis for the global burden of disease study 2017. Lancet.

[11] Hall MG, Garrett JJ, Barrington C (2014). La situacion economica: social determinants of contraceptive use in rural Honduras. Glob Public Health.

[12] Munakampe MN, Zulu JM, Michelo C. Contraception and abortion knowledge, attitudes and practices among adolescents from low and middle-income countries: a systematic review. BMC Health Serv Res. 2018;18(1). 10.1186/s12913-018-3722-5 PMC6267062 30497464

[13] Oussayef NL, Pillai SK, Honein MA (2016). Zika virus—10 public health achievements in 2016 and future priorities. MMWR Morb Mortal Wkly Rep.

[14] González Vélez AC, Diniz SG (2016). Inequality, Zika epidemics, and the lack of reproductive rights in Latin America. Reprod Health Matters.

[15] Tong A, Sainsbury P, Craig J (2007). Consolidated criteria for reporting qualitative research (COREQ): a 32-item checklist for interviews and focus groups. Int J Qual Health Care.

[16] Glaser B, Strauss A (1999). The discovery of grounded theory: strategies for qualitative research.

[17] Borges ALV, Moreau C, Burke A, Dos Santos OA, Chofakian CB (2018). Women's reproductive health knowledge, attitudes and practices in relation to the Zika virus outbreak in Northeast Brazil. PLoS One.

[18] Gomez HM, Mejia Arbelaez C, Ocampo Canas JA (2020). A qualitative study of the experiences of pregnant women in accessing healthcare services during the Zika virus epidemic in Villavicencio, Colombia, 2015-2016. Int J Gynaecol Obstet.

[19] Rios-Zertuche D, Blanco LC, Zuniga-Brenes P, Palmisano EB, Colombara DV, Mokdad AH (2017). Contraceptive knowledge and use among women living in the poorest areas of five Mesoamerican countries. Contraception..

[20] Speizer IS, Irani L, Barden-O'Fallon J, et al. Inconsistent fertility motivations and contraceptive use behaviors among women in Honduras. Reprod Health. 2009;6:19. 10.1186/1742-4755-6-19. 10.1186/1742-4755-6-19 PMC2783011 19925660

[21] Silva-Filho AL, Lira J, Rocha AL, Ferreira MC, Lamaita RM, Candido EB (2016). Non-hormonal and hormonal intrauterine contraception: survey of patients' perceptions in four Latin American countries. Eur J Contracept Reprod Health Care.

[22] Burgos-Munoz SJ, Toro-Huamanchumo CJ (2018). Zika knowledge and preventive practices among reproductive-age women from Lambayeque, Peru. Eur J Obstet Gynecol Reprod Biol.

[23] Musso D, Ko AI, Baud D (2019). Zika virus infection - after the pandemic. N Engl J Med.

[24] Burke A, Moreau C (2016). Family planning and Zika virus: the power of prevention. Semin Reprod Med.

[25] Madeiro AP, Rufino AC (2017). Maltreatment and discrimination in induced abortion care: perception of women in Teresina, state of Piaui, Brazil. Cien Saude Colet.

[26] Sebert Kuhlmann A, Shato T, Fu Q, Sierra M. Intimate partner violence, pregnancy intention and contraceptive use in Honduras. Contraception. 2019;100(2):137–41. 10.1016/j.contraception.2019.03.050. 10.1016/j.contraception.2019.03.050 30980825

[27] Lassi ZS, Musavi NB, Maliqi B, Mansoor N, de Francisco A, Toure K (2016). Systematic review on human resources for health interventions to improve maternal health outcomes: evidence from low- and middle-income countries. Hum Resour Health.

[28] Goldthwaite LM, Velasquez G (2016). Family planning and the Zika era. Curr Opin Obstet Gynecol.

[29] Nash E (2019). Abortion rights in peril - what clinicians need to know. N Engl J Med.

[30] Alonge O, Rodriguez DC, Brandes N, Geng E, Reveiz L, Peters DH (2019). How is implementation research applied to advance health in low-income and middle-income countries?. BMJ Glob Health.

