# Supplementary material for: Contraception and post abortion services: qualitative analysis of users’ perspectives and experiences following Zika epidemic in Honduras
Source: BMC Womens Health. 2020 Sep 12;20:199. doi: 10.1186/s12905-020-01066-7 (PMC7488691; doi:10.1186/s12905-020-01066-7)
Supplement: Supplementary file 1 — Additional file 1. [file 12905_2020_1066_MOESM1_ESM.docx]

**Semi-structured interviews for Post Abortion Care – accompanying men**

Note to interviewer: Begin the interview only if participants have signed the informed consent

| **A: Country: Honduras**  **B: City:** Tegucigalpa | **C: code:**  **D: Interview number:** |
| --- | --- |
| **E: Clinic/Hospital:** | **F: Date:** |
| **G: Interviewer:** | **:** |
| **Comments:** | |

**Post abortion Care**

*Introduction: Now, if you allow me, I would like to ask you about the reasons for your wife hospitalization.*

|  | **Open question** | **Probes** |
| --- | --- | --- |
| 1 | Who have you come to accompany? | What is your relationship with the woman who is staying? Couple, mother, brothe |
| 2 | Please tell me why the woman you are accompanying to the hospital today has come?  Has she been admitted to the post-abortion care room? | Ask if she is coming for an emergency or has already been treated and is returning for a follow-up. |
| 3 | Please tell me what happened to the woman since she started feeling sick and decided to come to the hospital - | Where was she? Who was with her?  - What happened to her? What symptoms did she have?  - How did you get to the hospital? How long did it take? |
| 4 | When you arrived at the hospital...  Where was she received to get admitted to the hospital?  Please tell were you able to go with her you? | - Did the hospital ask you to pay for her care?  - Were you asked to bring any items or medications needed for her care? |
| 5 | Do you know what treatment did she receive? | - Did they explain what treatment they were going to do? Did they have a chance to ask questions and get answers?  - When were you informed? |
| 6 | Are you satisfied with the care? Why? | - In general, did the health care team give you information about your questions and needs? |

Would you like to make any other comment? Thank you!
