# Supplementary material for: Contraception and post abortion services: qualitative analysis of users’ perspectives and experiences following Zika epidemic in Honduras
Source: BMC Womens Health. 2020 Sep 12;20:199. doi: 10.1186/s12905-020-01066-7 (PMC7488691; doi:10.1186/s12905-020-01066-7)
Supplement: Supplementary file 2 — Additional file 2. [file 12905_2020_1066_MOESM2_ESM.docx]

**Semi-structured interviews for Post Abortion Care – Women**

Note to interviewer: Begin the interview only if participants have signed the informed consent

| **A: Country: Honduras**  **B: City:** Tegucigalpa | **C: code:**  **D: Interview number:** |
| --- | --- |
| **E: Clinic/Hospital:** | **F: Date:** |
| **G: Interviewer:** | **:** |
| **Comments:** | |

**Post abortion Care**

*Introduction: Now, if you allow me, I would like to ask you about the reasons for your current hospitalization.*

|  | **Open question:** | **Probes** |
| --- | --- | --- |
| 1 | Please tell me why you have come to the hospital today?  Have you been admitted to the post-abortion care room? | Inquire about whether you are coming for an emergency or have already been treated and are returning for a follow-up. |
| 2 | Please tell me about the time you started feeling sick and decided to come to the hospital | - where were you? Who were with you?  - What happened to you? What symptoms did you have?  - How did you get to the hospital? How long did it take? How did you feel at that time?   - -Who accompanied you to the hospital? |
| 3 | When he arrived at the hospital...  Where did you go to be admitted to the hospital?  Please tell me what happens until you were admitted to the waiting room?  What happened during the time you were waiting in the waiting room? | Did a friend or family member accompany you?  - Did the hospital ask you to pay for your care?  - Were you asked to bring any items or medications needed for your care? |
| 4 | What treatment did you receive?  - Cleaning the Matrix  - To improve or remove pain  - Procedures:  - LUI (Matrix Scraping)  - MVA aspirated under local anesthesia (not asleep) - | - ¿Recuerda quién la atendió en la sala de cuidados post aborto?   Do you remember who treated you in the post-abortion care room?  - Did they explain what treatment they were going to do, did you have a chance to ask questions and get answers?  What did they tell you to do? |
| 5 | What were your post-discharge care instructions? | Where were you instructed to do your post-discharge monitoring? |
| 6 | - ¿Está satisfecha con la atención? ¿Por qué?   12 - Are you satisfied with the care you received? Why?  - | - In general, did the health care team pay attention to your questions and needs? - Did you feel comfortable? - Did you have privacy when you were seen? - How did the health care team treat you? |
| 7 | Did you receive planning counseling? Which one? | - Do you know how long after the abortion your body is ready to become pregnant again? - - Do you know how long to wait between pregnancies so that your health and your child's health are not affected? |
| 8 | Did you receive any family planning method? |  |

Would you like to make any other comment?

Thank you!
