# Supplementary material for: Contraception and post abortion services: qualitative analysis of users’ perspectives and experiences following Zika epidemic in Honduras
Source: BMC Womens Health. 2020 Sep 12;20:199. doi: 10.1186/s12905-020-01066-7 (PMC7488691; doi:10.1186/s12905-020-01066-7)
Supplement: Supplementary file 3 — Additional file 3. [file 12905_2020_1066_MOESM3_ESM.docx]

**FOCUS GROUP GUIDE WITH WOMEN/MEN**

Note to interviewer: Begin the interview only if participants have signed the informed consent

| **A: Country: Honduras**  **B: City:** Tegucigalpa | **C: Focus Group Number:**  **D: Total of participants:** |
| --- | --- |
| **E: Clinic/Hospital:** | **F: Date:** |
| **G: Interviewer:** | **H: Observer/secretary:** |
| **Comments:** | |

**Part I: Knowledge of Zika and Sources of Information**

Introduction: To begin I will ask you some questions regarding what you know or have heard about the Zika virus

|  | **Open question** | **Probes** |
| --- | --- | --- |
| 1 | What have you heard or what do you know about the Zika virus? - | - How can you get zika (causes)? - How can we perceive that someone is infected (signs and symptoms) - What are the problems, consequences and risks of contracting zika |
| 2 | What are the risk if a pregnant women gets Zika? | - For the mother - For the baby (Microcephaly, Malformations, Neurological diseases, Guillan Barre) |
| 3 | What have you heard or what do you know about dengue and chikungunya? | - - What is their relationship to zika? - - What are the differences between them? |
| 4 | What information did you receive at the clinic/hospital? | - Did you receive information at the health center/hospital? Who informed you? - Did you have access to brochures or materials distributed at the health center? - What do you think about these materials? |
| 5 | Beside that, how did you get informed about Zika? (sources of information) | - TV, Radio, Internet, Social networks, relatives or Friends? - What information do you find most credible or useful? |
| 6 | Do you think you have enough information? What else would you like to know? |  |

**Part II: Zika Virus Prevention**

Introduction: Now let's talk about the ways that can be implemented to prevent Zika

|  | **Open question** | **Probes** |
| --- | --- | --- |
| 7 | How can you prevent to get Zika virus? |  |
| 8 | How do you and the people you live with do to avoid being infected by the Zika virus? |  |
| 9 | Do the people who live in your neighborhood usually implement some actions to avoid getting infected by the Zika virus? Which ones? |  |
| 10 | What actions are usually implemented by public authorities to prevent infection with the Zika virus? |  |
| 11 | What difficulties, if any, have you had in taking prevention measures during the Zika epidemic? |  |
| 12 | If a woman is pregnant, what measures should she take to prevent infection by Zika? | Do you know of other ways to get Zika besides from a mosquito? Do you know that it can be transmitted through sex?  How can sexual transmission be prevented? |

**Part III: Sexual and reproductive health; needs related to sexual and reproductive health services and barriers to access**

|  | **Open questions** | | **Probes** |
| --- | --- | --- | --- |
| 13 | | Where do you go for pregnancy planning or contraception? |  |
| 14 | | What kind of contraceptive care or counseling do health centers or hospitals provide? |  |
| 15 | | What difficulties, if any, do you encounter in accessing contraceptive methods or sexual and reproductive health counseling? | - No family planning methods are available. - Privacy - Are not offered to you despite their existence - The spouse does not allow you to plan - No couples counseling - Abuse by staff - Shame/stigma |
| 16 | | What is your opinion regarding the different contraceptive methods? |  |
| 17 | | What are the things you like |  |
| 18 | | What do they like most and least about the care you receive at this health center/hospital? | - Treatment of patients by health care personnel. Did they feel respected, listened to? - Facilities: comfort and cleanliness of the place. Privacy |
| 19 | | If you were the director of this health center/hospital, what would you do to improve sexual and reproductive health service? That is, in terms of contraception and family planning | - - Do you think the health care staff could have done more for you? What and why? - - What would you recommend to improve the facilities where you received care (comfort, cleanliness, privacy, decoration, lighting, room temperature or ventilation)? - -What would you recommend regarding the organization of waiting time, opening hours for the different procedures, etc |

20. Would you like to make any additional comment?

Thank you very much for participating in this group.
